# Precision cardiovascular risk prediction in type 1 diabetes: An IMI2 SOPHIA analysis

**DOI:** 10.1038/s41467-026-72029-z

**Published:** 2026-04-16

**Authors:** Sofia Pazmino, Stefanie Schmid, Jordi Blanch, Daniel Coral, Nele Steenackers, Nicole Forestier, Laura Gallardo-Nuell, Naichuan Zhang, Birgit Reinhart-Steininger, Yenny Leal, Amar van Laar, Rafael Ramos, Shreekar Bharadwaj Araveti, Ditte F. Heidemann, Kinga Nowak, Jonathan Rosen, Carmen Hurtado del Pozo, Christian-Dominik Möller, Chantal Mathieu, Carel W. le Roux, Paul W. Franks, Jose Manuel Fernandez-Real, Stefanie Lanzinger, Bart Van der Schueren

**Affiliations:** 1 Clinical and Experimental Endocrinology, Department of Chronic Diseases and Metabolism, KU Leuven, Leuven, Belgium; 2 Institute of Epidemiology and Medical Biometry, University of Ulm, Ulm, Germany; 3 German Center for Diabetes Research (DZD), Munich-Neuherberg, Germany; 4 Grup Investigació en Salut Vascular de Girona (ISV- Girona), Institut Universitari d’Investigació en Atenció Primària Jordi Gol (IDIAP Jordi Gol), Catalunya, Spain; 5 Network for Research on Chronicity, Primary Care, Prevention and Health Promotion (RICAPPS), Girona, Spain; 6 Genetic and Molecular Epidemiology Unit, Lund University Diabetes Centre, Department of Clinical Science, Lund University, Helsingborg, Sweden; 7 Department of Nutrition and Movement Sciences, Research Institute of Nutrition and Translational Research in Metabolism, Maastricht University, Maastricht, The Netherlands; 8 Diabetes Centre Bad Aibling, Bad Aibling, Germany; 9 Nutrition, Eumetabolism and Health Group, Institut d’Investigació Biomèdica de Girona (IDIBGI-CERCA), Girona, Spain; 10 CIBER Centro de Investigación Biomédica en Red de Fisiopatología de la Obesidad y Nutrición (CIBERobn), Instituto de Salud Carlos III, Madrid, Spain; 11 Department of Diabetes, Endocrinology and Nutrition, Dr. Josep Trueta University Hospital, Girona, Spain; 12 Clinical Division of Endocrinology and Metabolism, Department of Medicine III, Medical University of Vienna, Vienna, Austria; 13 Grup Investigació en Salut Vascular de Girona (ISV- Girona), Institut d’Investigació Biomèdica de Girona (IdIBGi), Catalunya, Spain; 14 Departament de Ciències Mèdiques, Universitat de Girona, Catalunya, Spain; 15 Serveis d’Atenció Primària, Girona, Institut Català de Salut, Catalunya, Spain; 16 Novo Nordisk A/S, Copenhagen, Denmark; 17 University College Dublin School of Medicine, Dublin, Ireland; 18 Breakthrough T1D (former JDRF), New York, NY USA; 19 Bürgerhospital Frankfurt, Frankfurt am Main, Germany; 20 Department of Endocrinology, University Hospitals Leuven, Leuven, Belgium; 21 Diabetes Complications Research Centre, University College Dublin, Dublin, Ireland; 22 PHURI, Queen Mary University of London, London, UK

**Keywords:** Type 1 diabetes, Obesity, Diabetes complications

## Abstract

Cardiovascular disease (CVD) is a major long-term complication and the leading cause of morbidity and mortality among individuals with type 1 diabetes (T1D), with a substantially higher prevalence compared to the general population and driven by multiple interrelated risk factors—underscoring the urgent need for accurate risk assessment. To support more tailored approaches to CVD prevention, recently, five discordant phenotypic risk profiles for CVD were identified in the general population in Europe with diverse relationship between body mass index and cardiometabolic biomarkers. Here, we show their applicability in 44,212 people with T1D. Improved glycemic control was linked to a decrease in CVD risk as people with T1D with lower glycated hemoglobin belonged to the baseline concordant cluster. This supports the contention that glycemic control in people with T1D is an integral part of lowering CVD risk.

## Abstract (web summary)

Risk profiling based on how BMI interacts with cardiovascular markers was useful in the general population. In type 1 diabetes— where cardiovascular risk is already high— these profiles are notably valuable for tailored approaches as they reveal how high glucose may hide other risk factors

## Introduction

People living with type 1 diabetes (T1D) have a higher risk for developing cardiovascular disease (CVD), resulting in a decreased life-expectancy of about 10 years when compared to the general population^1,2^. CVD assessment in T1D is challenging—not only do traditional risk factors like hypertension and dyslipidemia apply, but chronic hyperglycemia also plays a role. However, the extent of its detrimental effect remains unclear, as CVD risk remains elevated even with good glycemic control^2,3^. Therefore, the observation that weight gain is on the raise in people living with T1D is of great concern^4,5^, as it may further increase their CVD risk. In order to facilitate precision prevention of CVD, Coral et al.^6^ identified five discordant profiles in the general population, where cardiometabolic risk differs from what is typically expected based on body mass index (BMI). In this context, each discordant profile represents a phenotypic subgroup whose biomarker levels are either higher or lower than expected for peers of the same age, sex, smoking status, and BMI. Each individual is assigned a probability of belonging to each phenotype, reflecting the continuous nature of these classifications. Here, we aim to briefly put the study of Coral et al.^6^ into the perspective of people living with T1D to explore how overweight and obesity worsen their already elevated CVD risk.

## Results

### Study design, population and outcomes

International/multi-center cross-sectional data from adults ( ≥ 18 years) living with T1D, followed between 2010 and 2022, were used for this replication study. Data sources were the University Hospital Leuven (KUL) in Belgium^4^, the German/Austrian/Luxembourgian/Swiss diabetes prospective (DPV) follow-up registry^7^ and the Jordi Gol I Gurina Foundation (SIDIAP)^8^ in Spain. The same biomarkers traditionally associated with CVD risk were included: age, sex, smoking, BMI, fasting glucose, lipids (HDL, LDL, triglycerides), blood pressure, serum creatinine, ALT, CRP, and waist/hip ratio. The first visit with the most information available for all biomarkers was used. To maximize data completeness, we used a six-month time window before and after the visit, based on the most available information. For each individual, missing values were first filled using their own observations within this time window; if no within-person data were available, imputation was then performed using information from similar participants in the UK Biobank using a k-nearest neighbors algorithm. KUL included a population of 2241 individuals (51% female, 49% male), DPV included 25,422 individuals (47% female, 53% male), and SIDIAP had a population of 16,549 (43% female and 57% male) used in this analysis (Table 1).

**Table 1:** Baseline characteristics across cohorts

	Female			Male		
	KUL (*n* = 1133)	SIDIAP (*n* = 7080)	DPV (*n* = 11993)	KUL (*n* = 1108)	SIDIAP (*n* = 9469)	DPV (*n* = 13429)
Age (years)	45.0 [29.5–59.0]	42 [32–57]	25.2[18.6–50.3]	44.0 [30.0–57.0]	42 [33–53]	23.0 [18.5–49.8]
Disease duration (years)	19.0 [9.0–32.0]	6.9 [2.2 − 9.7]	11.9 [6.8–19.8]	17.0 [7.0–30.0]	6.4 [2.0 − 9.0]	11.1 [5.9–19.1]
BMI (kg/m2)	24.5 [22.2–28.0]	24.8 [22.1 − 28.5]	24.60[22.1–28.0]	24.9 [22.6–27.5]	25.3 [22.9 − 28.1]	24.4 [22.0–27.4]
Smoking (n, %)	67 (8.9%)	1 801 (25.4%)	1695 (14.1%)	120 (15.8%)	3 335 (35.2%)	2578 (19.2%)
HbA1c (%)	7.60 [7.0–8.3]	7.8 [7.0 − 8.8]	7.7 [7.0–8.8]	7.7 [7.0–8.2]	7.8 [7.0 − 8.9]	7.7 [6.9–8.7]
Fasting glucose (mmol/L)	8.4 [6.8–10.4]	8.2 [5.8 − 11.6]	9.3 [7.9–11.2]	8.3 [6.8–10.1]	8.3 [5.9 − 11.8]	9.3 [7.8–11.1]
HDL (mmol/L)	1.8 [1.5–2.2]	1.6 [1.3 − 1.9]	1.7 [1.4–2.0]	1.5 [1.2–1.7]	1.3 [1.1 − 1.6]	1.4 [1.2–1.6]
LDL (mmol/L)	2.0 [1.7–2.5]	2.7 [2.2 − 3.2]	2.7 [2.2–3.3]	2.0 [1.6–2.5]	2.7 [2.2 − 3.2]	2.6 [2.0–3.2]
Triglycerides (mmol/L)	1.1 [0.8–1.3]	0.9 [0.7 − 1.3]	1.1 [0.8–1.6]	1.1 [0.8–1.4]	1.0 [0.8 − 1.5]	1.1 [0.8–1.7]
C-reactive protein (mg/L)	2.1 [0.9–5.1]	4.2 [1.5 − 8.9]	0.9 [0.2–5.0]	1.2 [0.6–3.4]	3.7 [1.4 − 10]	0.6 [0.2–1.5]
Serum creatinine (μmol/L)	65.4 [59.2–74.3]	64.3 [55.7 − 74.3]	65.4 [57.5-75.2]	80.9 [73.4–90.2]	80.0 [70.7 − 92.0]	79.6 [70.7–89.3]
ALT (U/L)	15.0 [13.0–17.3]	12.0 [0.0 − 19.0]	16.2 [13.0–22.0]	21.0 [16.0–25.0]	16.0 [0.0 − 25.0]	21.0 [16.0–29.0]
Systolic blood pressure (mmHg)	130.0 [120.0–140.0]	122 [112 − 134]	124.0 [116.5–133]	133.0 [126.0–142.0]	129 [120 − 137]	130.0 [120.0–139.0]
Diastolic blood pressure (mmHg)	75.8[70.0–80.0]	72 [66 − 79]	75.0 [70.0–80.0]	78.0 [71.0–82.0]	75 [69 − 80]	76.0 [70.0–80.5]
Waist/hip ratio	NA	NA	0.9 [0.8–09]	NA	NA	0.9 [0.9–1.0]

Data presented as median and [interquartile ranges] or frequency and percentage.

*NA* not available, *KUL* University Hospital Leuven in Belgium, *SIDIAP* Jordi Gol I Gurina Foundation in Spain, *DPV* German/Austrian/Luxembourgian/Swiss diabetes prospective follow-up registry.

### Risk profiles

**Fig. 1:**
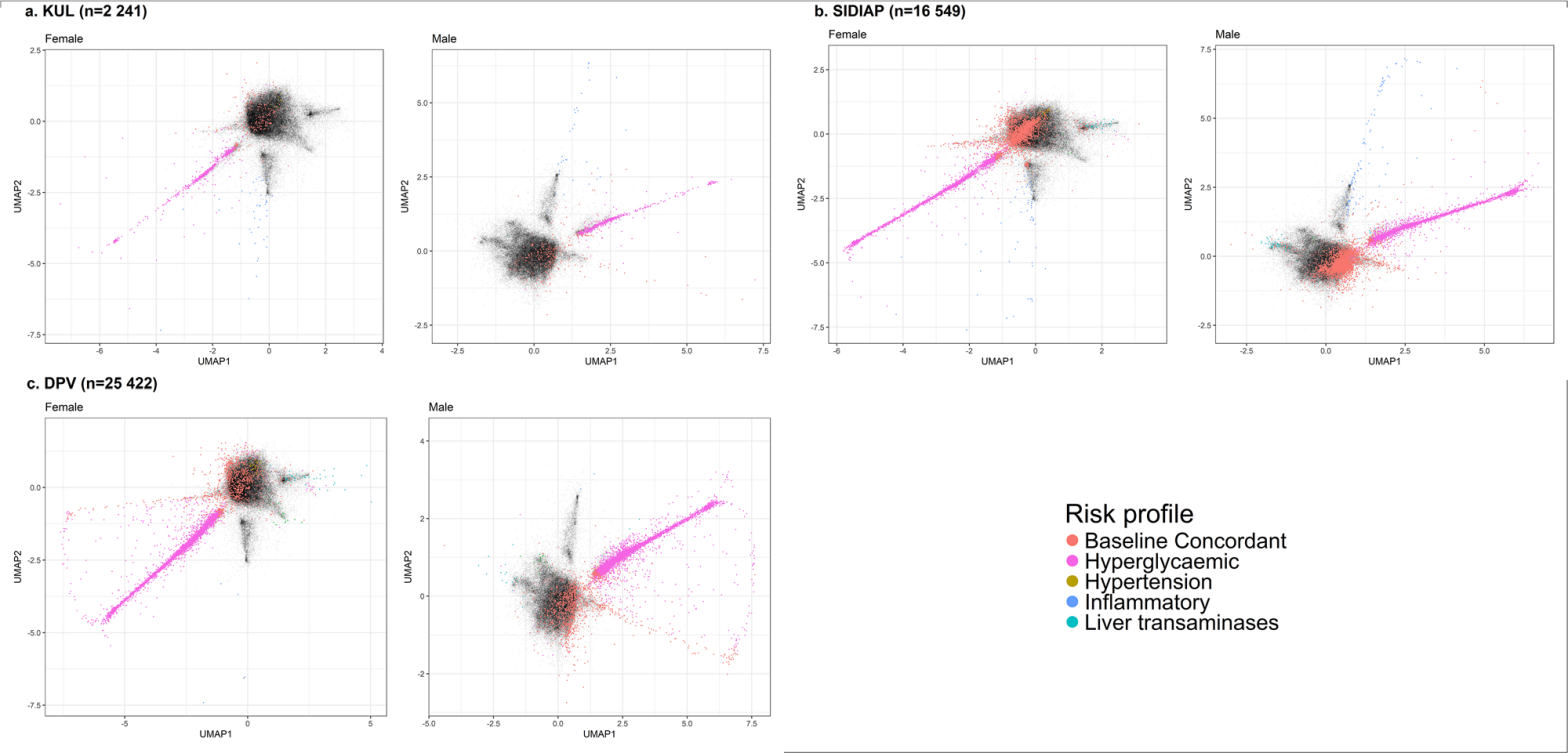
Concordant and discordant phenotypic risk profi les in type 1 diabetes. Uniform Manifold Approximation and Projection for Dimension Reduction (UMAP) 2D projections: the population with type 1 diabetes from **a** KU Leuven, **b** DPV and **c** SIDIAP are in colors projected on the UK Biobank general population in black.

An overview of the analysis pipeline has been previously published^6,9^. Briefly, to replicate the identified subgroups^6^, we calculated discordant scores based on the expected population-level associations between BMI and each biomarker, adjusted for age and smoking, generated from the UK Biobank (UKB). We then computed allocation probabilities into the concordant and discordant profiles described by Coral et al.^6^, and visualized these allocations in relation to the concordant and discordant profiles using the Uniform Manifold Approximation and Projection (UMAP) embedding. Three risk profiles were mostly represented in the population with T1D: concordant, hyperglycemic, and inflammatory (Fig. 1). The majority of individuals (55-76%) belonged to the discordant hyperglycemic profile (Table 2), while this cluster was the smallest ( ~ 2.5%) in the general population analysis done by Coral et al.^6^. As could be expected, in the population with T1D, mean HbA1c% was significantly lower (*p*-value < 0.0001, t-test per cohort per sex) in the concordant cluster (mean ± standard deviation 7.3 ± 1.5/7.3 ± 1.1 in KUL, 7.4 ± 1.8/7.2 ± 1.7 in DPV, and 7.6 ± 1.4/7.5 ± 1.4 in SIDIAP for females/males, respectively) than in the hyperglycemic profile (7.9 ± 1.2/7.9 ± 1.1 in KUL, 8.2 ± 1.7/8.1 ± 1.6 in DPV, and 8.3 ± 1.7/8.4 ± 1.7 in SIDIAP for females/males respectively).

**Table 2:** Risk profile distribution across cohorts

Sex	Risk profile	Overall			Below 60 years of age			Above 60 years of age		
		KUL (*n* = 1133/1108)	SIDIAP (7080/9469)	DPV (11993/13429)	KUL (*n* = 868/881)	SIDIAP (5562/7943)	DPV (10138/11611)	KUL (265/227)	SIDIAP (1518/1516)	DPV (1855/1818)
Female	Hyperglycaemic	762 (67.2%)	3485 (54.3%)	10514 (87.7%)	605 (69.7%)	2946 (53.0%)	9004 (88.8%)	157 (59.2%)	899 (59.2%)	1510 (81.4%)
	Baseline concordant	307 (27.1%)	3002 (42.4%)	1342 (11.2%)	215 (24.8%)	2430 (43.7%)	1012 (10.0%)	91 (34.4%)	575 (37.9%)	330 (17.8%)
	Hypertension	17 (1.5%)	81 (1.1%)	53 (0.4%)	15 (1.7%)	77 (1.4%)	50 (0.5%)	2 (0.8%)	4 (0.3%)	3 (0.2%)
	Liver transaminases	2 (0.2%)	59 (0.8%)	48 (0.4%)	1 (0.1%)	45 (0.8%)	41 (0.4%)	1 (0.4%)	11 (0.7%)	7 (0.4%)
	Adverse lipid	-	9 (0.1%)	28 (0.2%)		7 (0.1%)	24 (0.2%)	-	2 (0.1%)	4 (0.2%)
	Inflammatory	45 (4.0%)	84 (1.2%)	7 ( < 0.1%)	31 (3.6%)	58 (1.0%)	6 ( < 0.1%)	14 (5.3%)	27 (1.8%)	1 ( < 0.1%)
Male	Hyperglycaemic	703 (63.5%)	4979 (52.6%)	11616 (86.5%)	580 (65.8%)	4191 (52.7%)	10254 (88.3%)	123 (54.2%)	785 (51.8%)	1362 (74.9%)
	Baseline concordant	362 (32.7%)	4288 (45.3%)	1754 (13.1%)	270 (30.6%)	3619 (45.5%)	1311 (11.3%)	92 (40.5%)	671 (44.3%)	444 (24.4%)
	Liver transaminases	3 ( < 0.1%)	70 (0.7%)	31 (0.2%)	3 (0.3%)	57 (0.7%)	25 (0.2%)	-	13 (0.9%)	6 (0.3%)
	Adverse lipid	8 (0.7%)	10 (0.1%)	25 (0.2%)	5 (0.6%)	7 (0.1%)	20 (0.2%)	3 (1.3%)	3 (0.2%)	5 (0.3%)
	Inflammatory	32 (2.9%)	123 (1.3%)	3 ( < 0.1%)	23 (2.6%)	79 (1.0%)	2 ( < 0.1%)	9 (4.0%)	43 (2.8%)	1 ( < 0.1%)

Data presented as frequency and (percentage)

*KUL* University Hospital Leuven in Belgium, *SIDIAP* Jordi Gol I Gurina Foundation in Spain, *DPV* German/Austrian/Luxembourgian/Swiss diabetes prospective follow-up registry.

### Prediction models

Same as Coral et al.^6^, we compared two types of sex-specific survival prediction models: one based solely on the previously mentioned biomarkers with variables and interactions used in the current CVD risk stratification tool endorsed by the European Society of Cardiology (SCORE2)^10,11^ and another that also included profile allocation probabilities, to assess whether adding profile information would improve the prediction of major adverse cardiac events (MACE). Table 3 contains the event rates (MACE, macrovascular complications, retinopathy, and nephropathy) across cohorts. Through comparison of nested models, we showed that adding profile allocation probabilities improved the predictive capability of some of these models (significant likelihood ratio tests, considered to be the gold-standard)^12,13^: macrovascular complications in the KUL cohort for males, MACE in the SIDIAP cohort for males, the extended MACE for females, and retinopathy in the KUL -males- and DPV -females- cohorts (Supplementary Data 1). This improvement was also reported for MACE by Coral et al.^1^, particularly for men in UKB. Other risk engines specifically for diabetes, like the United Kingdom Prospective Diabetes Study (UKPDS)^14^ and even T1D-specific engines such as STENO-T1D^15^ have been shown to underestimate the risk of CVD in T1D^14^ or do not include BMI^15^. Nevertheless, the newer LIFE-T1D^16^ risk score that contains similar variables to SCORE2 with more focus on kidney health (albuminuria), concomitant complications (retinopathy), and age of T1D diagnosis has considered BMI as an important predictor.

**Table 3:** Event rates across cohorts

Event	Age stratification	Female			Male		
		KUL	SIDIAP	DPV	KUL	SIDIAP	DPV
MACE	Overall	155 (13.7%)	905 (12.8%)	176 (1.5%)	179 (16.1%)	1339 (14.1%)	278 (2.1%)
MACE	≤60 years at baseline	60(6.9%)	326 (5.9%)	72 (0.7%)	71 (8.1%)	755 (9.5%)	140 (1.2%)
MACE	>60 years at baseline	95 (35.8%)	579 (38.1%)	104 (5.6%)	108 (47.6%)	584 (38.5%)	138 (7.6%)
MACE + TIA + angina	Overall	208 (18.4%)	1129 (15.9%)	195 (1.6%)	226 (20.4%)	1540 (16.3%)	296 (2.2%)
MACE + TIA + angina	≤60 years at baseline	84 (9.7%)	542 (9.7%)	83 (0.8%)	94 (10.7%)	950 (11.9%)	156 (1.3%)
MACE + TIA + angina	>60 years at baseline	124 (46.8%)	587 (38.7%)	112 (6.0%)	132 (58.2%)	555 (36.6%)	140 (7.7%)
Nephropathy	Overall	356 (31.4%)	222 (3.1%)	948 (7.9%)	294 (26.5%)	424 (4.5%)	1030 (7.7%)
Nephropathy	≤60 years at baseline	192 (22.1%)	98 (1.8%)	685 (6.8%)	157 (17.8%)	238 (3.0%)	776 (6.7%)
Nephropathy	>60 years at baseline	164 (61.9%)	148 (9.7%)	263 (14.2%)	137 (60.4%)	186 (12.3%)	254 (14.0%)
Retinopathy	Overall	350 (30.9%)	190 (2.7%)	619 (5.2%)	357 (32.2%)	306 (3.2%)	668 (5.0%)
Retinopathy	≤60 years at baseline	217 (25.0%)	167 (3.0%)	429 (4.2%)	229 (26.0%)	232 (2.9%)	500 (4.3%)
Retinopathy	>60 years at baseline	133 (50.2%)	41 (2.7%)	190 (10.2%)	128 (56.4%)	74 (4.9%)	168 (9.2%)

*MACE* major adverse cardiovascular event (stroke, myocardial infarction and all-cause death), *TIA* transient ischemic attack, *KUL* University Hospital Leuven in Belgium, *SIDIAP* Jordi Gol I Gurina Foundation in Spain, *DPV* German/Austrian/Luxembourgian/Swiss diabetes prospective follow-up registry.

For KUL, macrovascular events instead of MACE and macrovascular events + death in the extended definition of MACE.

Data presented as frequency and percentage.

### Net benefit

From a clinical point of view and considering the potential benefit for the population, statistically significant improvement should not be the only consideration when choosing one tool over another. Net benefit analysis allows for a better translation of the benefits and harms for decision making. Many medical decisions involve balancing trade-offs, such as treating patients to prevent disease-related harm while minimizing the risk of treatment side effects, or diagnosing patients with a disease while avoiding unnecessary testing for healthy individuals. Net benefit is a decision-analytic measure that is increasingly used to quantify both benefits and harms on a common scale. To assess the additional net benefit of discordant profiles, decision curves were used to evaluate whether implementing interventions to prevent CVD would be beneficial (Supplementary Data 2). Coral et al.^6^ showed that models with and without discordant profile data, generally performed better than no intervention or universal intervention across various MACE probability thresholds up to 15%. It also showed that adding profile information at a 10% 10-year MACE risk threshold resulted in an average net benefit of 4 additional correct interventions and 37 additional unnecessary interventions correctly avoided per 10,000 individuals tested^3^. In a population with T1D, at the same threshold of 10%, the net benefit for MACE in men in SIDIAP was 2 additional correct interventions while correctly avoiding 5,746 unnecessary interventions for every 10,000 individuals tested (Supplementary Data 2). Especially for T1D, this could be a way forward to aid decision-making on CVD prevention, since it adds no burden to the healthcare system by using already commonly measured biomarkers and because the risk profile seems more impactful in T1D than in the general population.

### T1D-specific relationships

Finally, to explore how the relationship of BMI–biomarker discordance in this population with T1D differs from the general population, sex-specific linear models for the residuals were fitted while adjusting for age, smoking status and disease duration before clustering. In terms of starting points (intercept), the main differences were present in fasting glucose for both males and females. Additionally, in females, differences in systolic blood pressure were seen, while in males, LDL was also found to be altered (Supplementary Data 3). These biomarkers could prove interesting targets for reducing CVD risk in people living with T1D with potentially greater effects than in the general population.

## Discussion

This expansion study leverages robust, large-scale, multi-centre data to validate an established risk profiling framework in a new, clinically significant population, and the inclusion of decision curve analysis further enhances its translational relevance and potential for clinical adoption. Importantly, to define the phenotypic risk profiles, commonly used biomarkers routinely collected during standard medical consultations are used, meaning that the proposed approach would not add any data collection or workload burden for healthcare practitioners. With the exception of the waist-to-hip ratio, which remains relatively easy to implement and could be feasibly adopted in future research and/or clinical practice. However, a way of clinically translating the measured biomarkers into the risk profiles is needed, especially by integrating a user-friendly free calculator (https://shiny.gbiomed.kuleuven.be/UMAP_app/) into daily clinical practice, ideally linked to electronic health record systems. Additionally, while the study demonstrated improved CVD and retinopathy prediction in certain populations with T1D, substantial increases in C-statistics are inherently challenging to achieve in already well-calibrated models. Therefore, even small gains in discrimination and decision-curve outcomes can have meaningful implications at the population level, particularly for the early identification and prevention of complications in T1D. On the other hand, the cross-sectional allocation of risk profiles raises uncertainty about their temporal stability, while the stability of the identified phenotypic profiles over time opens the opportunity for future research, as longitudinal analyses could provide valuable insights into the temporal consistency of these profiles not only in a population with T1D but also in the general population. Nevertheless, emerging evidence suggests that cross-sectional information can, in some cases, offer predictive performance comparable to—or even exceeding—that of longitudinal data^17,18^ while also being more feasible for use in routine clinical practice, which is why we believe that this current study still presents benefits to clinical practice. Also, our findings may have limited generalizability given the reliance on predominantly European data with relatively little ethnic heterogeneity and variation in health care systems.

While the risk profiles identified by Coral et al.^6^ are well-suited for the general population, as expected, there is an overexpression of the hyperglycemic profile in individuals with T1D. Furthermore, better glycemic control (HbA1c) was associated with a lower CVD risk profile, such as the baseline concordant cluster, which more closely aligns with the general population. This highlights the critical importance of maintaining glycemic control in T1D management^19^ as a key factor in effective CVD risk control. Also, it contradicts previous findings that better glycemic control does not lower CVD^20^. On the other hand, our results point to chronic hyperglycemia masking other relevant risk profiles or biomarkers in people living with T1D, further hinting at different pathways to CVD depending on glycemic control^3^ and mediated by traditional risk factors over time -disease duration^21,22^. Future research in T1D would benefit from considering models based not only on typical CVD risk factors but also approaches that account for the impact of glucose control before allocating/clustering into risk profiles. It could also explore the use of longitudinal data to further validate BMI- subclassifications and their evolution in diverse cohorts. Understanding the heterogeneity of diabetes will be a key step towards precision medicine^23^, particularly for CVD prevention in T1D.

## Methods

This research complies with all relevant ethical regulations, per study cohort the committee and institution that approved have been named.

### Study cohorts

#### University Hospital Leuven (KUL)

Patient data were obtained from the type 1 diabetes (T1D) registry of University Hospital Leuven^4^, a tertiary care center located in the Flemish Brabant region, northern Belgium. All individuals with a clinical diagnosis of T1D who received care at this hospital were eligible for inclusion in the registry. A confirmed diagnosis by a healthcare provider was required. Ethical approval was acquired from the local ethics committee for the ethical conduct of human research (Ethics Committee Research UZ/KU Leuven). Written informed consent was obtained from the participants.

#### German/Austrian/Luxembourgian/Swiss diabetes prospective (DPV) follow-up registry

This is a multicenter database initiated in Germany in 1995 to standardize the documentation of demographic and diabetes-related clinical data from specialized care centers and has now expanded to include centers in Austria, Luxembourg, and Switzerland^7^. Clinical and demographic data are entered locally using the standardized DPV electronic health record system. Participating centers submit anonymized data biannually to Ulm University, Germany, where they undergo plausibility checks. Any discrepancies identified are returned to the clinics for clarification or correction prior to data consolidation. The anonymized dataset is utilized for clinical research and quality monitoring purposes (www.d-p-v.eu). The DPV initiative has received ethical approval from the ethics committee of Ulm University, as well as the respective review boards of all participating institutions^7^. Written informed consent was obtained from the participants.

#### Jordi Gol I Gurina Foundation (SIDIAP)

The Information System for Research in Primary Care (SIDIAP)^8^ is a comprehensive database of electronic health records from primary care, designed to support healthcare research. It compiles data from 328 primary care centers operated by the Catalan Health Institute across Catalonia, Spain. Since 2006, SIDIAP has collected pseudo-anonymized health records for over 8 million individuals, covering approximately 75% of the Catalan population. This project was approved by the Scientific and Ethical Committee of “IDIAP Jordi Gol” and the Institute of Biomedical Research of Girona, Dr. Josep Trueta (IDIBGI). Written informed consent was obtained from the participants.

### Statistical analysis

#### Data Preparation

Same as Coral et al.^6^, we included 13 biomarkers across all cohorts: fasting glucose (FG) in mmol/l; lipid fractions (HDL, LDL, triglycerides) in mmol/l; systolic and diastolic blood pressure in mmHg; serum creatinine in μmol/l; alanine aminotransferase (ALT) in U/l; C-reactive protein (CRP) in mg/l; waist-to-hip ratio in cm; age in years; smoking status (1 = current smoker, 0 = non-smoker); and sex (male/female). Units were harmonized across datasets. No BMI thresholds were applied. Outliers exceeding 5 standard deviations from the mean were excluded prior to analysis. All analyses were stratified by sex to account for known gender-related differences in BMI, biomarkers, and cardiometabolic risk.

#### UMAP projection and profile identification

This cluster analysis follows a multi-step analytical pipeline^6^ that combines linear modeling, residual extraction, dimensionality reduction, and unsupervised clustering to examine biomarker data. The main goals are to evaluate the relationship between a set of biomarkers and BMI (adjusting for additional covariates), and to identify latent subgroups based on the residual patterns from these models. The analysis is conducted separately for males and females.

Biomarker residuals were projected into two dimensions using the umap function from the R package uwot (v0.1.16)^24^, with nearest neighbor count set as a function of cohort size. The parameter binary_edge_weights was set to TRUE, implementing PacMAP, a UMAP variant that better preserves both global and local data structures. Additionally, we used dens_scale = 1 to enable densMAP, which enhances density preservation. Clusters were identified by constructing a proximity network and applying the Leiden community detection algorithm after seeding with the leading eigenvector method (igraph, v2.0.2)^25^. The algorithm, run over 500 iterations, optimized modularity through node movement, partition refinement, and network aggregation. Eigen centrality scores were calculated per individual and used to determine cluster centres and covariance matrices within a Gaussian mixture model. To account for the instability of central clusters, a concordant distribution (zero mean, identity covariance) was added. Individuals with high overlap between discordant and concordant profiles were assigned to the latter, improving cluster specificity. The final model provided both hard cluster assignments and profile probability scores for each individual.

#### Model comparisons

We used likelihood ratio tests and changes in C-statistics to compare nested models. Variance explained by discordant profiles was quantified using likelihood-based methods, unaffected by threshold choices. We also performed decision curve analyses to assess clinical utility, quantifying net benefit (true positives) and net interventions avoided (true negatives) across varying probability thresholds, both per cohort and pooled across studies^26^.

#### Predictive value of profiles

To assess added predictive value, we compared nested regression models with and without discordant profile information. Using profile allocation probabilities in regression models is problematic due to their sum-to-one constraint. Coral et al.^6^ addressed this by using the log-contrast approach from compositional data analysis, using the concordant profile as reference. For each individual, discordant profile probabilities were divided by the concordant probability, and their natural logarithms were used as predictors.

#### Nested Cox regression models

We evaluated the association between profile allocations and risk of MACE using nested Cox proportional hazards models over 10-year follow-up periods. The predictors included variables from the SCORE2 algorithm (validated in diabetic populations), (see Supplementary Data 1). Individuals with pre-existing CVD were excluded.

#### Phenotypic discordance with BMI in T1D

We examined the association between BMI and each biomarker, adjusting for age, and smoking status, and disease duration, using linear regression models (see Supplementary Data 3). Comparison of regression coefficients was performed by using the Clifford Clogg et al. formula (1995)^27^.

### Reporting summary

Further information on research design is available in the Nature Portfolio Reporting Summary linked to this article.

## Supplementary information

Description Of Additional Supplementary File

Supplementary data 1-3

Reporting Summary

Transparent Peer Review file

## Source data

Source data

## Data Availability

**KUL**. The raw KUL data -individual-level data- are protected and are not available due to patient protection and data privacy laws. Upon request for research purposes, aggregated data that support the findings of this study can be made available. Requests should be sent to the corresponding author (Prof. Bart Van der Schueren - bart.vanderschueren@uzleuven.be) with an expected response within a month of the request. **DPV**. The datasets generated during and/or analyzed during the current study are not publicly available due to data protection and data use rules of the DPV initiative, but aggregated data are available upon request from the lead of the DPV initiative (PD Dr. Stefanie Lanzinger, stefanie.lanzinger@uni-ulm.de) with an expected response within a month of request. Data on the individual level cannot be shared because of patient protection and the agreements provided by the patient. **SIDIAP**. Any researcher is able to request SIDIAP data to conduct a study. A five-step procedure takes place before data access is granted: (i) the researcher(s) must send an application (standardized form available at www.sidiap.org and study protocol) to the SIDIAP team; (ii) the application is approved by SIDIAP’s Scientific Committee which evaluates the scientific quality and feasibility of the proposal; (iii) the study protocol is approved by the Clinical Research Ethics Committee of IDIAPJGol; (iv) the principal investigator of the study must sign a Good Practice form and, in some cases, an agreement between parties is needed; and (v) a meeting between the research team and the SIDIAP team is arranged to discuss the procedures and set the data extraction. Further information is available online (https://www.sidiap.org/index.php/menu-solicitudesen/application-proccedure) or by contacting Anna Moleras (sidiap@idiapjgol.org). Data access is limited to researchers from public organizations and collaboration with private institutions is possible when a study is required by a regulatory agency or for non-commercial studies within a European project financed by the European Commission. In accordance with current European and national law, the data used in this study are only available for the researchers participating in this study. Thus, we are not allowed to distribute or make publicly available the data to other parties. The datasets generated and analysed for this study are not publicly available due to legal and ethical restrictions related to data privacy but could be made available upon request to Rafel Ramos (rramos.girona.ics@gencat.cat) with an expected response within a month of request and a maximum time of use of five years afterwards a new request should be submitted. Source data are provided with this paper.

## Code availability

All analyses were performed in programming language R v.4.4 (https://cran.r-project.org/bin/windows/base/old/4.4.0/)^28^. The scripts used can be found at https://github.com/danielcoral/SOPHIA_Cross_Sectional.^9^ UMAP app available via this link: https://shiny.gbiomed.kuleuven.be/UMAP_app/.
