# Supplementary material for: Precision cardiovascular risk prediction in type 1 diabetes: An IMI2 SOPHIA analysis
Source: Nat Commun. 2026 Apr 16;17:5239. doi: 10.1038/s41467-026-72029-z (PMC13261115; doi:10.1038/s41467-026-72029-z)
Supplement: Supplementary file 1 — Description Of Additional Supplementary File [file 41467_2026_72029_MOESM1_ESM.pdf]

Description of Additional supplementary files

Supplementary Data 1. Model comparison without and with profile allocation probabilities.

Supplementary Data 2. Decision curves for MACE progression at a 10% threshold comparing models with and without profile information

Supplementary Data 3. Linear model estimates from UK Biobank and its comparison to the population with T1D from KUL, DPV and SIDIAP
