# Supplementary material for: Precision cardiovascular risk prediction in type 1 diabetes: An IMI2 SOPHIA analysis
Source: Nat Commun. 2026 Apr 16;17:5239. doi: 10.1038/s41467-026-72029-z (PMC13261115; doi:10.1038/s41467-026-72029-z)
Supplement: Supplementary file 4 — Transparent Peer Review file [file 41467_2026_72029_MOESM4_ESM.pdf]

nature portfolio

Peer Review File

Precision cardiovascular risk prediction in type 1 diabetes: An IMI2 SOPHIA analysis

Corresponding Author: Professor Bart Van der Schueren

This file contains all reviewer reports in order by version, followed by all author rebuttals in order by version.

Version 0:

Reviewer comments:

Reviewer #2

(Remarks to the Author)
This study by Pazmino et al. addresses an important gap in the field of diabetes precision medicine by extending risk profiling frameworks, originally developed for type 2 diabetes and general populations, to individuals with type 1 diabetes (T1D). Given the substantial clinical need for better tools to predict complications in T1D, this work is timely and of high relevance.
Using large, multi-center European datasets (KUL, DPV, SIDIAP) comprising over 24,000 individuals with T1D, the authors validate the applicability of five previously defined discordant risk profiles. They report a notably higher prevalence of the discordant hyperglycemic (DHG) profile (55–76% in T1D vs. 2.5% in the general population) and an elevated prevalence of the discordant inflammatory (DIS) profile (15–22%). Conversely, patients with better glycemic control tend to cluster in the baseline concordant (BC) profile, with lower HbA1c levels (6.9–7.6%) compared to those in the DHG profile (7.8–8.3%). Importantly, the addition of these phenotypic profiles to conventional survival models enhances the accuracy of predicting major adverse cardiovascular events (MACE). The study highlights two aspects of clinical utility: (1) it reinforces the evidence that optimal glycemic control is associated with a lower risk of complications; (2) it demonstrates, through decision curve analysis, that the phenotypic model can reduce overtreatment, avoiding approximately 1,287 unnecessary interventions per 10,000 patients at a 10% 10-year MACE risk threshold in the SIDIAP cohort.
The strenghts oft he study ist he robust, large-scale, multi-center data, which builds upon and validates an established risk profiling framework in a new, clinically significant population. The inclusion of decision curve analysis strengthens the translational relevance and supports potential clinical adoption.

However, the study also has some weaknesses, While the paper clearly shows improved prediction, more discussion on how these profiles could be operationalized in routine care (e.g., integration into clinical workflows or EHR systems) would enhance practical impact and the prevalence estimates could benefit from stratification by age, disease duration, or treatment regimen to explore whether these factors influence profile distribution.

Overall, Pazmino et al. present a significant and well-executed study that extends the promise of precision medicine to people living with T1D. The work addresses an unmet need and offers a clear pathway for improving complication risk stratification in this population. With some additional discussion of clinical implementation and pathophysiological underpinnings, this paper could serve as a valuable reference for both researchers and clinicians.

Major comments
1. The study appropriately follows the methodological framework established by Coral et al., including: (i) defining phenotypic profiles based on BMI-biomarker residuals to identify discordant phenotypes; (ii) applying the same dimensionality reduction and clustering approach (UMAP combined with Leiden clustering) with comparable parameter settings; and (iii) validating the model performance using C-statistics to assess the added predictive value of phenotypic information. However, in extending this framework to individuals with T1D, the authors incorporated disease duration as an additional covariate. It remains unclear whether the impact of T1D-specific treatments — particularly insulin therapy and other glucose-lowering agents — was accounted for. Did all patients with T1D use insulin, or where also other treatments used?

2. In the DPV registry, only individuals aged >35 years were included in the analysis. It would strengthen the paper to clearly report the full age distribution across all cohorts and to analyze whether age impacts the distribution of phenotypic profiles.

For example, stratifying the sample by age groups (e.g., <50 years vs. ≥50 years) could reveal whether certain discordant profiles are more prevalent in older patients, which may have implications for generalizability and clinical interpretation.

3. The rationale for selecting an upper risk threshold of “up to 25%” for the decision curve analysis should be clearly stated, particularly since Coral et al. used a threshold of 15%. Providing justification for this difference — such as differences in event rates, risk distribution, or clinical practice considerations in T1D — would clarify the clinical relevance and comparability of the decision curve results.

4. It would be valuable to clarify whether additional microvascular complications, such as nephropathy (e.g., urinary albumin-to-creatinine ratio) or retinopathy, were considered as outcomes or incorporated into the phenotypic stratification. Given that microvascular complications are highly relevant in T1D, including these could potentially refine the risk profiles and enhance prediction accuracy.

5. Although Supplemental Table 2 demonstrates improved prediction using 10-year follow-up data, the phenotypic profiles themselves are derived from cross-sectional information. The manuscript should explicitly acknowledge that the stability of these phenotypes over time was not assessed, if this is the case. Highlighting this as a limitation would provide a more balanced interpretation and help guide future research on the temporal consistency of discordant profiles in T1D populations.

Minor comments

6. Although the term “discordant profiles” is introduced alongside the appropriate reference, it would enhance clarity for readers if the concept were briefly defined and described within the main text. A concise explanation of what constitutes a discordant profile and its clinical relevance would help orient readers who may not be familiar with the prior framework.

7. Supplemental Table 3 presents linear model parameters (intercepts and slopes for BMI, age, and smoking) comparing the general UK Biobank population and the three T1D cohorts (KUL, DPV, SIDIAP) to highlight biomarker differences. While significant differences are indicated in bold, adding an explicit P-value column would provide clearer statistical context and allow readers to interpret the significance more directly.

8. Consider adding a detailed table summarizing the baseline characteristics of each cohort, including key metabolic factors (e.g., BMI, HbA1c), diabetes duration, treatment regimens, and other relevant variables. This would improve transparency and allow readers to better understand the context and comparability of the study populations.

9. Figures: The quality of Figures 2 should be improved to ensure that all text and labels are fully legible at standard viewing sizes. Figure Legends: Provide more detailed figure legends for Figures 1 and 2, clearly explaining all elements, colors, and abbreviations used. The current annotations are insufficient and limit interpretability, particularly for readers less familiar with the clustering and risk profile terminology.

Reviewer #3

(Remarks to the Author)
I co-reviewed this manuscript with one of the reviewers who provided the listed reports. This is part of the Nature Communications initiative to facilitate training in peer review and to provide appropriate recognition for Early Career Researchers who co-review manuscripts.

Reviewer #4

(Remarks to the Author)
This is a very well written and interesting article, but would benefit from a more nuanced discussion of the magnitude of the results and what these mean. Specific comments:

- It is mentioned missing data from the first visit was imputed using information from similar participants. Could you have used data from future visits of people with missing data (in addition to non-missing first visit data) to impute missing information from their first visit instead? This is likely to be a better reflection than using data from other participants
- It would be useful to add in 95% confidence intervals around the measures in the results tables
- Please add into the tables the number of events that occurred during the follow-up time in each cohort
- Interpretation – most of the results are very small, e.g. change in C statistic range from -0.012 to 0.004 (depending on the cohort) and mostly appear to be statistically insignificant (although confidence intervals would be useful to report) so it is difficult to conclude there is any benefit. Although it is difficult to achieve large changes in C-indices, the results from the decision curve analysis are also small finding an additional 2 people with T1D per 10,000 would be treated appropriately with the additional phenotype profile data at a risk threshold of 10%. These results suggest very minimal benefit at a population level of adding in profile data into risk prediction and this needs to be balanced against the additional work that would be involved for a clinician to collect and asses these additional data. The paper would be improved by a better discussion of these results.
- A brief discussion of the limitations would be useful.

Version 1:

Reviewer comments:

Reviewer #2

(Remarks to the Author)
hank you for your detailed, point-by-point rebuttal. The revisions and additional analyses substantially clarify the manuscript, and I find that the previously raised concerns have been thoroughly addressed.

Reviewer #3

(Remarks to the Author)
I co-reviewed this manuscript with one of the reviewers who provided the listed reports. This is part of the Nature Communications initiative to facilitate training in peer review and to provide appropriate recognition for Early Career Researchers who co-review manuscripts.

Reviewer #4

(Remarks to the Author)
Thank you to the authors for the changes to the paper - the manuscript is greatly improved especially with the changes to the discussion of how the results are interpreted.

However, I do have some remaining concerns over the interpretation of the findings in relation to predictive performance. There are two statements that seem to overstate the results. The first is: "Through comparison of nested models, we showed that adding profile allocation probabilities improved the predictive capability of some of these models (macrovascular complications, MACE and retinopathy), reflected by significant likelihood ratio tests and difference in C-statistics (Supplemental Table 3)” and the second statement is: "Additionally, while the study demonstrates improved CVD and retinopathy prediction..."

For the mast majority of outcomes and data sources, there is no evidence of differences in the C-statistics or LRT. Stating that the predictive capabilities of the models for macrovascular complications, MACE and retinopathy is improved seems to be overstating what is shown in the data, where there is some statistically significant change in the LRT or C-statistic for these outcomes, but only for some cohorts and in some sexes (and even then, the magnitude of the 'improvement' is very small). Given there is an increased likelihood of having a false positive result when more tests are run, there is little evidence, when considering the entirety of the results, of any improvements in prediction. Care should be taken not to pick out the few instances where these outcomes are improved (only in certain cohorts or sexes) and implying that these apply across the outcomes. E.g. it is suggested that predictive performance of MACE is improved but this comes only from the likelihood ratio test being very slightly improved in one cohort (SIDIAP) for males only. It ignores that there is no evidence of a change in LRT for females in the SIDIAP cohort, or for males or females in the DPV cohort, and no evidence of change in C statistics for MACE across any of these cohorts or sexes.

Version 2:

Reviewer comments:

Reviewer #4

(Remarks to the Author)
Thank you to the authors for addressing these comments and updating the paper. Your revisions have aligned the findings in the text much more explicitly with the results tables, and the discussion is substantially improved. All my previous concerns have been addressed.

Open Access This Peer Review File is licensed under a Creative Commons Attribution 4.0 International License, which permits use, sharing, adaptation, distribution and reproduction in any medium or format, as long as you give appropriate credit to the original author(s) and the source, provide a link to the Creative Commons license, and indicate if changes were made.
In cases where reviewers are anonymous, credit should be given to 'Anonymous Referee' and the source.
The images or other third party material in this Peer Review File are included in the article's Creative Commons license, unless indicated otherwise in a credit line to the material. If material is not included in the article's Creative Commons license and your intended use is not permitted by statutory regulation or exceeds the permitted use, you will need to obtain permission directly from the copyright holder.
To view a copy of this license, visit https://creativecommons.org/licenses/by/4.0/

January 2026

Dear Reviewers,

We greatly appreciate your input on our paper. Your valuable suggestions have greatly helped us in fine-tuning the manuscript and running insightful additional analyses. We thank the reviewers for suggesting that we should take clinical implementation more into account, for this we have developed a software application that enables clinicians to assign individuals to the corresponding risk profiles. We feel this will greatly facilitate implementation in clinical practice. We have adapted the manuscript as outlined in the following point-by-point comment section and included additional individuals and ran additional analyses to respond to your comments. Furthermore, there is now a more thorough description and discussion of our limitations. We hope this will clarify and address all the concerns. Please find our responses below -in blue-.

We ensure that this article contains only original work. The material in this manuscript has not been published before and is not being considered for publication elsewhere in whole or in part in any language. The conflicts of interest are reported and the copyright assignment form and other required documents will be forthcoming.

On behalf of all authors.
Yours Sincerely,

Bart Van der Schueren, MD, PhD, Prof.
Clinical and Experimental Endocrinology,
Department of Chronic diseases, Metabolism and Ageing
KU Leuven, Belgium

UZ LEUVEN

KU LEUVEN

<u>Reviewers' comments:</u>

Reviewer #2 (Remarks to the Author):

This study by Pazmino et al. addresses an important gap in the field of diabetes precision medicine by extending risk profiling frameworks, originally developed for type 2 diabetes and general populations, to individuals with type 1 diabetes (T1D). Given the substantial clinical need for better tools to predict complications in T1D, this work is timely and of high relevance.

Using large, multi-center European datasets (KUL, DPV, SIDIAP) comprising over 24,000 individuals with T1D, the authors validate the applicability of five previously defined discordant risk profiles. They report a notably higher prevalence of the discordant hyperglycemic (DHG) profile (55–76% in T1D vs. 2.5% in the general population) and an elevated prevalence of the discordant inflammatory (DIS) profile (15–22%). Conversely, patients with better glycemic control tend to cluster in the baseline concordant (BC) profile, with lower HbA1c levels (6.9–7.6%) compared to those in the DHG profile (7.8–8.3%).

Importantly, the addition of these phenotypic profiles to conventional survival models enhances the accuracy of predicting major adverse cardiovascular events (MACE). The study highlights two aspects of clinical utility: (1) it reinforces the evidence that optimal glycemic control is associated with a lower risk of complications; (2) it demonstrates, through decision curve analysis, that the phenotypic model can reduce overtreatment, avoiding approximately 1,287 unnecessary interventions per 10,000 patients at a 10% 10-year MACE risk threshold in the SIDIAP cohort.

The strengths of the study is the robust, large-scale, multi-center data, which builds upon and validates an established risk profiling framework in a new, clinically significant population. The inclusion of decision curve analysis strengthens the translational relevance and supports potential clinical adoption.

However, the study also has some weaknesses, While the paper clearly shows improved prediction, more discussion on how these profiles could be operationalized in routine care (e.g., integration into clinical workflows or EHR systems) would enhance practical impact and the prevalence estimates could benefit from stratification by age, disease duration, or treatment regimen to explore whether these factors influence profile distribution.

Response:

Thank you very much for your thoughtful and encouraging feedback. Your supportive comments reinforce our commitment to continuing this line of research to improve clinical prediction and a more holistic approach to the clinical care for people living with T1D.

However, the study also has some weaknesses, While the paper clearly shows improved prediction, more discussion on how these profiles could be operationalized in routine care (e.g., integration into clinical workflows or EHR systems) would enhance practical impact and the prevalence estimates could benefit from stratification by age, disease duration, or treatment regimen to explore whether these factors influence profile distribution.

Response:

As our analyses uses biomarkers that are routinely collected in daily clinical practice, we feel that it will be easy to implement in regular clinical care without creating additional burden to the health care system or health care practitioners. We do agree that incorporation in electronic health records the best way to help clinicians make a more informed decision. Therefore we

have created a free calculator (https://shiny.gbiomed.kuleuven.be/UMAP_app/) has been create to help in clinically translating the measured biomarkers into the risk profiles, this calculator can allocate any new individual into their respective risk profile given the different measurements. This paper will be the reference for future implementation exercises, but it is also of prime importance that our findings are disseminated in the public domain.

Changes to manuscript are highlighted in track changes

Overall, Pazmino et al. present a significant and well-executed study that extends the promise of precision medicine to people living with T1D. The work addresses an unmet need and offers a clear pathway for improving complication risk stratification in this population. With some additional discussion of clinical implementation and pathophysiological underpinnings, this paper could serve as a valuable reference for both researchers and clinicians.

Response:
We have incorporated a more well-rounded discussion on limitations and translation into clinical practice. We truly appreciate your recognition of the importance and relevance of our work. Our goal has been to help advance precision medicine approaches for individuals with type 1 diabetes, and it is gratifying to know that our efforts to extend and adapt existing risk profiling frameworks resonate with the needs of the field.

Changes to manuscript are highlighted in track changes

Major comments

1. The study appropriately follows the methodological framework established by Coral et al.[1], including: (i) defining phenotypic profiles based on BMI-biomarker residuals to identify discordant phenotypes; (ii) applying the same dimensionality reduction and clustering approach (UMAP combined with Leiden clustering) with comparable parameter settings; and (iii) validating the model performance using C-statistics to assess the added predictive value of phenotypic information. However, in extending this framework to individuals with T1D, the authors incorporated disease duration as an additional covariate. It remains unclear whether the impact of T1D-specific treatments — particularly insulin therapy and other glucose-lowering agents — was accounted for. Did all patients with T1D use insulin, or where also other treatments used?

Response:
As part of our definition for including individuals with type 1 diabetes, they had to be on insulin and on no other medication for diabetes except for metformin. We have now gone back to the DPV registry and included 220 people (93 females and 127 males) on GLP-1 or SGLT-2 at baseline and performed a sensitivity analysis in this subpopulation which did not differ from the overall population.

Changes to manuscript are highlighted in track changes

2. In the DPV registry, only individuals aged >35 years were included in the analysis. It would strengthen the paper to clearly report the full age distribution across all cohorts and to analyze whether age impacts the distribution of phenotypic profiles. For

example, stratifying the sample by age groups (e.g., <50 years vs. ≥50 years) could reveal whether certain discordant profiles are more prevalent in older patients, which may have implications for generalizability and clinical interpretation.

Response:
We have now included individuals with type 1 diabetes aged 18 years and older from the DPV and SIDIAP registry. In addition, we have added tables on cluster distribution and event rates stratified by age category. We selected a cut-off of 60 years based on the findings of Patsoukaki et al.[2], who reported that type 1 diabetes is associated with a higher overall risk of cardiovascular disease events and all-cause mortality compared to type 2 diabetes, particularly in individuals older than 60 years.

Changes to manuscript are highlighted in track changes

3. The rationale for selecting an upper risk threshold of "up to 25%" for the decision curve analysis should be clearly stated, particularly since Coral et al. used a threshold of 15%. Providing justification for this difference — such as differences in event rates, risk distribution, or clinical practice considerations in T1D — would clarify the clinical relevance and comparability of the decision curve results.

Response:

4. The choice of 25% was two-fold, one because it has been shown that individuals with T1D have a significantly higher risk of cardiovascular disease (CVD) compared to the general population and also because the prevalence was higher in our cohorts than what was seen in Coreal, D et al's paper[1]. Moreover, we have corrected for several CVD risk factor of as well as diabetes duration since longer diabetes duration has been shown to be a main contributing factor for higher risk found in people with T1D compared even to type 1 diabetes versus type 2 diabetes, especially in the over 60 years of age. [2] We have included as Supplemental Table 2 all the event rates.It would be valuable to clarify whether additional microvascular complications, such as nephropathy (e.g., urinary albumin-to-creatinine ratio) or retinopathy, were considered as outcomes or incorporated into the phenotypic stratification. Given that microvascular complications are highly relevant in T1D, including these could potentially refine the risk profiles and enhance prediction accuracy.

Response:
We recognize that microvascular complications are highly important in a population with type 1 diabetes. We have now expanded our scope to include them and the analyses have been added to the manuscript. In particular, retinopathy prediction is improved by our model.

Changes to manuscript are highlighted in track changes

5. Although Supplemental Table 2 demonstrates improved prediction using 10-year follow-up data, the phenotypic profiles themselves are derived from cross-sectional information. The manuscript should explicitly acknowledge that the stability of these phenotypes over time was not assessed, if this is the case. Highlighting this as a limitation would provide a more balanced interpretation and help guide future research on the temporal consistency of discordant profiles in T1D populations.

Response:
While the stability of the identified phenotypic profiles over time was not assessed in the present study, which we have now explicitly acknowledged as a limitation, it does open the opportunity for future research. Longitudinal analyses could provide valuable insights into the temporal consistency of these profiles not only on a population with type 1 diabetes but also in the general population. Nevertheless, emerging evidence suggests that cross-sectional information can, in some cases, offer predictive performance comparable to—or even exceeding—that of longitudinal data,[3,4] while also being more feasible for use in routine clinical practice which is why we believe that this current study presents benefits to clinical practice.

"(…) On the other hand, the cross-sectional allocation of risk profiles raises uncertainty about their temporal stability, while the stability of the identified phenotypic profiles over time opens the opportunity for future research as longitudinal analyses could provide valuable insights into the temporal consistency of these profiles not only on a population with T1D but also in the general population. Nevertheless, emerging evidence suggests that cross-sectional information can, in some cases, offer predictive performance comparable to—or even exceeding—that of longitudinal data[3,4] while also being more feasible for use in routine clinical practice which is why we believe that this current study still presents benefits to clinical practice."

Minor comments

6. Although the term "discordant profiles" is introduced alongside the appropriate reference, it would enhance clarity for readers if the concept were briefly defined and described within the main text. A concise explanation of what constitutes a discordant profile and its clinical relevance would help orient readers who may not be familiar with the prior framework.

Response:
To be added to the first paragraph on the main text:

"People living with type 1 diabetes (T1D) have a higher risk for developing cardiovascular disease (CVD), resulting in a decreased life-expectancy of about 10 years when compared to the general population.[5,6] CVD assessment in T1D is challenging—not only do traditional risk factors like hypertension and dyslipidemia apply, but chronic hyperglycemia also plays a role. However, the extent of its detrimental effect is still unclear, as CVD risk remains elevated even when good glycemic control is achieved.[6,7] Therefore, the observation that weight gain is on the raise in people living with T1D is of great concern,[8,9] as it may further increase their CVD risk. In order to facilitate precision prevention of CVD, Coral et al[1] identified five discordant profiles in the general population, where cardiometabolic risk differs from what is typically expected based on body mass index (BMI). In this context, each discordant profile represents a phenotypical subgroup whose biomarker levels are either higher or lower than expected compared to peers of the same age, sex, smoking status, and BMI. Each individual is assigned a probability of belonging to each phenotype, reflecting the continuous nature of these classifications. Here, we aim to briefly put the study of Coral et al[1] into the perspective of people living with T1D to explore how overweight and obesity worsens their already elevated CVD risk."

7. Supplemental Table 3 presents linear model parameters (intercepts and slopes for BMI, age, and smoking) comparing the general UK Biobank population and the three T1D cohorts (KUL, DPV, SIDIAP) to highlight biomarker differences. While significant differences are indicated in bold, adding an explicit P-value column would provide clearer statistical context and allow readers to interpret the significance more directly.

Response:
This is an excellent suggestion that will improve the clarity of the results. A p-value column has now been added to Supplemental Table 5 to provide clearer statistical context for the coefficient differences between cohorts and to facilitate interpretation of their significance.

8. Consider adding a detailed table summarizing the baseline characteristics of each cohort, including key metabolic factors (e.g., BMI, HbA1c), diabetes duration, treatment regimens, and other relevant variables. This would improve transparency and allow readers to better understand the context and comparability of the study populations.

Response:
To enhance transparency and interpretability of the study populations, we have now included a detailed baseline characteristics table (Supplemental Table 1) per cohort, including the key biomarkers used, diabetes duration, and other relevant clinical variables. This addition provides a clearer overview of cohort comparability and contextualizes the observed differences across populations.

9. Figures: The quality of Figures 2 should be improved to ensure that all text and labels are fully legible at standard viewing sizes. Figure Legends: Provide more detailed figure legends for Figures 1 and 2, clearly explaining all elements, colors, and abbreviations used. The current annotations are insufficient and limit interpretability, particularly for readers less familiar with the clustering and risk profile terminology.

Response:
The figures have been regenerated in high-resolution SVG format to ensure that all text, labels, and visual elements are fully legible at standard viewing sizes. Additionally, the figure legends have been substantially revised to provide more detailed explanations of all elements, colour schemes, and abbreviations used. These revisions aim to improve clarity and interpretability, particularly for readers who may be less familiar with the clustering and risk profile terminology.

Reviewer #3 (Remarks to the Author):

I co-reviewed this manuscript with one of the reviewers who provided the listed reports. This is part of the Nature Communications initiative to facilitate training in peer review and to provide appropriate recognition for Early Career Researchers who co-review manuscripts.

Response:
Thank you for informing us, and we appreciate the transparency regarding the co-review process. We commend the Nature Communications initiative to involve and formally recognize Early Career Researchers in peer review, as it promotes valuable mentorship and capacity

building within the scientific community. We are grateful to both reviewers for their time, expertise, and thoughtful evaluation of our work.

Reviewer #4 (Remarks to the Author):

This is a very well written and interesting article, but would benefit from a more nuanced discussion of the magnitude of the results and what these mean.

Response:
Thank you very much for your positive feedback and for highlighting this important point. We appreciate your suggestion to include a more nuanced discussion of the magnitude of the results and their implications. In response, we have a dedicated paragraph on strengths and limitations for a clearer interpretation of the effect sizes. I believe this addition strengthens the paper by offering a more comprehensive understanding of the results and their significance.

Specific comments:

- It is mentioned missing data from the first visit was imputed using information from similar participants. Could you have used data from future visits of people with missing data (in addition to non-missing first visit data) to impute missing information from their first visit instead? This is likely to be a better reflection than using data from other participants

Response:
To maximize data completeness, we used a time window of six months before and six months after the visit with the most available information. For each individual, missing values were first filled using their own observations within this time window; if no within-person data were available, imputation was then performed using information from similar participants from UK Biobank by means of nearest neighbour. This has now been clarified in the manuscript:

- It would be useful to add in 95% confidence intervals around the measures in the results tables.
Response:
We apologize, it was not entirely clear to us to which results the reviewer was referring to. We have added 95% confidence intervals to the table on model comparisons for C-statistics (Supplementary Table 3) while the other tables have stayed with interquartile ranges. Please if further modification is needed we are more than open to them.

- Please add into the tables the number of events that occurred during the follow-up time in each cohort
Response:
We have added a table on event rates, also stratified by age category (Supplementary Table 2).

- Interpretation – most of the results are very small, e.g. change in C statistic range from -0.012 to 0.004 (depending on the cohort) and mostly appear to be statistically insignificant (although confidence intervals would be useful to report) so it is difficult to conclude there is any benefit. Although it is difficult to achieve large changes in C-indices, the results from the decision curve analysis are also small finding an additional 2 people with T1D per 10,000 would be treated appropriately with the additional phenotype profile data at a risk threshold of 10%. These

results suggest very minimal benefit at a population level of adding in profile data into risk prediction and this needs to be balanced against the additional work that would be involved for a clinician to collect and asses these additional data. The paper would be improved by a better discussion of these results.

Response:
We have added this as part of our discussion:
"(…) while the study demonstrates improved CVD and retinopathy prediction, substantial increases in C-statistics are inherently challenging to achieve in already well-calibrated models. Therefore, even small gains in discrimination and decision-curve outcomes can have meaningful implications at the population level, particularly for the early identification and prevention of complications in T1D."

- A brief discussion of the limitations would be useful.

Response:

We have incorporated a more well-rounded discussion on study strengths and weaknesses as well as the limitations for translation into clinical practice:

"This expansion study leverages robust, large-scale, multi-centre data to validate an established risk profiling framework in a new, clinically significant population, and the inclusion of decision curve analysis further enhances its translational relevance and potential for clinical adoption. Importantly, to define the phenotypic risk profiles, commonly used biomarkers routinely collected during standard medical consultations are used, meaning that the proposed approach would not add any data collection or workload burden for healthcare practitioners. With the exception of waist-to-hip ratio but that remains relatively easy to implement and could be feasibly adopted in future research and/or clinical practice. However, a way of clinically translating the measured biomarkers into the risk profiles is needed, especially by integrating a user-friendly free calculator (https://shiny.gbiomed.kuleuven.be/UMAP_app/) into daily clinical practice, ideally linked to electronic health record systems. Additionally, while the study demonstrates improved CVD and retinopathy prediction, substantial increases in C-statistics are inherently challenging to achieve in already well-calibrated models. Therefore, even small gains in discrimination and decision-curve outcomes can have meaningful implications at the population level, particularly for the early identification and prevention of complications in T1D. On the other hand, the cross-sectional allocation of risk profiles raises uncertainty about their temporal stability, while the stability of the identified phenotypic profiles over time opens the opportunity for future research as longitudinal analyses could provide valuable insights into the temporal consistency of these profiles not only on a population with T1D but also in the general population. Nevertheless, emerging evidence suggests that cross-sectional information can, in some cases, offer predictive performance comparable to—or even exceeding—that of longitudinal data[3,4] while also being more feasible for use in routine clinical practice which is why we believe that this current study still presents benefits to clinical practice. Also, our findings may have limited generalizability given the reliance on predominantly European data with relatively little heterogeneity in ethnicity and health care systems."

References:

1. Coral DE, Smit F, Farzaneh A, et al. Subclassification of obesity for precision prediction of cardiometabolic diseases. *Nature Medicine* 2024.
2. Patsoukaki V, Lind L, Lampa E, Radhi S, Eeg-Olofsson K, Eliasson B, Eriksson JW. Risk differences and underlying factors of cardiovascular events and mortality in patients with type 2 diabetes versus type 1 diabetes: a longitudinal cohort study of Swedish nationwide register data. *The Lancet Diabetes & Endocrinology* 2025; **13**(10): 848-62.
3. Rindfleisch A, Malter AJ, Ganesan S, Moorman C. Cross-Sectional versus Longitudinal Survey Research: Concepts, Findings, and Guidelines. *Journal of Marketing Research* 2008; **45**(3): 261-79.
4. Shmatko A, Jung AW, Gaurav K, et al. Learning the natural history of human disease with generative transformers. *Nature* 2025.
5. Ahuja A, Agrawal S, Acharya S, Reddy V, Batra N. Strategies for Cardiovascular Disease Prevention in Type 1 Diabetes: A Comprehensive Review. *Cureus* 2024; **16**(8): e66420.
6. Vergès B. Cardiovascular disease in type 1 diabetes, an underestimated danger: Epidemiological and pathophysiological data. *Atherosclerosis* 2024; **394**: 117158.
7. Miller RG, Orchard TJ, Costacou T. 30-Year Cardiovascular Disease in Type 1 Diabetes: Risk and Risk Factors Differ by Long-term Patterns of Glycemic Control. *Diabetes Care* 2022; **45**(1): 142-50.
8. Al Ozairi E, Steenackers N, Pazmino S, et al. Prevalence of obesity in people with and without type 1 diabetes across Belgium, Kuwait, and Mexico: an IMI2 SOPHIA study. *eClinicalMedicine* 2024; **77**: 102869.
9. Conway B, Miller RG, Costacou T, Fried L, Kelsey S, Evans RW, Orchard TJ. Temporal patterns in overweight and obesity in Type 1 diabetes. *Diabet Med* 2010; **27**(4): 398-404.

February 2026

Dear Reviewers,

We greatly appreciate your input on our paper. Your valuable suggestions greatly helped fine-tuning the manuscript and we are pleased that the revisions were well received.
We have addressed the remaining concerns over the interpretation of the findings in relation to predictive performance. Please find our responses below -in blue-.

We ensure that this article contains only original work. The material in this manuscript has not been published before and is not being considered for publication elsewhere in whole or in part in any language. The conflicts of interest are reported and the copyright assignment form and other required documents will be forthcoming.

On behalf of all authors.
Yours Sincerely,

Bart Van der Schueren, MD, PhD, Prof.
Clinical and Experimental Endocrinology,
Department of Chronic diseases, Metabolism and Ageing
KU Leuven, Belgium

UZ LEUVEN

KU LEUVEN

Reviewers' comments:

Reviewer #2 (Remarks to the Author):

Thank you for your detailed, point-by-point rebuttal. The revisions and additional analyses substantially clarify the manuscript, and I find that the previously raised concerns have been thoroughly addressed.

Response:
Thank you for your thoughtful assessment. We are glad to learn that the revisions and expanded analyses have resolved the earlier concerns. Your careful constructive feedback throughout this process has been invaluable.

Reviewer #3 (Remarks to the Author):

I co-reviewed this manuscript with one of the reviewers who provided the listed reports. This is part of the Nature Communications initiative to facilitate training in peer review and to provide appropriate recognition for Early Career Researchers who co-review manuscripts.

Response:
We are grateful to both reviewers for their time, expertise, and thoughtful evaluation of our work. We commend the Nature Communications initiative to involve and formally recognize Early Career Researchers in peer review, as it promotes valuable mentorship and capacity building within the scientific community.

Reviewer #4 (Remarks to the Author):

Thank you to the authors for the changes to the paper - the manuscript is greatly improved especially with the changes to the discussion of how the results are interpreted.

Response:
We greatly appreciate the time you have taken to evaluate the updated manuscript. Please find below our answer to your raised concerns in interpretation of the findings.

However, I do have some remaining concerns over the interpretation of the findings in relation to predictive performance. There are two statements that seem to overstate the results. The first is: "Through comparison of nested models, we showed that adding profile allocation probabilities improved the predictive capability of some of these models (macrovascular complications, MACE and retinopathy), reflected by significant likelihood ratio tests and difference in C-statistics (Supplemental Table 3)" and the second statement is: "Additionally, while the study demonstrates improved CVD and retinopathy prediction..."

For the vast majority of outcomes and data sources, there is no evidence of differences in the C-statistics or LRT. Stating that the predictive capabilities of the models for macrovascular complications, MACE and retinopathy is improved seems to be overstating what is shown in the data, where there is some statistically significant change in the LRT or C-statistic for these outcomes, but only for some cohorts and in some sexes (and even then, the magnitude of the 'improvement' is very small). Given there is an increased likelihood of having a false positive result when more tests are run, there is little evidence, when considering the entirety of the

results, of any improvements in prediction. Care should be taken not to pick out the few instances where these outcomes are improved (only in certain cohorts or sexes) and implying that these apply across the outcomes. E.g. it is suggested that predictive performance of MACE is improved but this comes only from the likelihood ratio test being very slightly improved in one cohort (SIDIAP) for males only. It ignores that there is no evidence of a change in LRT for females in the SIDIAP cohort, or for males or females in the DPV cohort, and no evidence of change in C statistics for MACE across any of these cohorts or sexes.

Response:

We have modified our text to explicitly include only the comparisons that showed a difference based on the likelihood ratio test (LRT). LRT is considered the gold-standard as a measure of added value in the predictive sense which not only measures improvement in discrimination - same as C-statistics- but also differences in calibration and with more power.[1,2] Furthermore, we agree that there is an increased likelihood of having a false positive result when more tests are run, which is why we had already incorporated a 5% false discovery rate to our findings to correct for multiple testing.

Moreover, especially from a clinical point of view and considering the potential benefit for the population, statistical significant improvement should not be the only consideration when choosing one tool over another, net benefit analysis allows for a better translation of the benefit and harms for decision making.

Modified text:

Same as Coral et al.[3], we compared two types of sex-specific survival prediction models: one based solely on the previously mentioned biomarkers with variables and interactions used in the current CVD risk stratification tool endorsed by the European Society of Cardiology (SCORE2) [4,5] and another that also included profile allocation probabilities, to assess whether adding profile information would improve the prediction of major adverse cardiac events (MACE). Through comparison of nested models, we showed that adding profile allocation probabilities improved the predictive capability of some of these models (significant likelihood ratio tests, considered to be the gold-standard)[1,2]: macrovascular complications in the KUL cohort for males, MACE in the SIDIAP cohort for males, the extended MACE for females, and retinopathy in the KUL -males- and DPV -females- cohorts (Supplemental Table 3). This improvement was also reported for MACE by Coral et al[1], particularly for men in UKB. Other risk engines specifically for diabetes, like the United Kingdom Prospective Diabetes Study (UKPDS)[6] and even T1D-specific engines such as STENO-T1D[7] have been shown to underestimate the risk of CVD in T1D[6] or do not include BMI.[7] Nevertheless, the newer LIFE-T1D[8] risk score that contains similar variables to SCORE2 with more focus on kidney health (albuminuria), concomitant complications (retinopathy) and age of T1D diagnosis has considered BMI as an important predictor.

Moreover, especially from a clinical point of view and considering the potential benefit for the population, statistical significant improvement should not be the only consideration when choosing one tool over another, net benefit analysis allows for a better translation of the benefit and harms for decision making. Many medical decisions involve balancing trade-offs, such as treating patients to prevent disease-related harm while minimizing the risk of treatment side effects, or diagnosing patients with a disease while avoiding unnecessary testing for healthy

individuals. Net benefit is a decision-analytic measure that is increasingly used to quantify both benefits and harms on a common scale. To assess the additional net benefit of discordant profiles, decision curves were used to evaluate whether implementing interventions to prevent CVD would be beneficial (Supplemental Table 4). Coral et al[3] showed that models with and without discordant profile data, generally performed better than no intervention or universal intervention across various MACE probability thresholds up to 15%. It also showed that adding profile information at a 10% 10-year MACE risk threshold resulted in an average net benefit of 4 additional correct interventions and 37 additional unnecessary interventions correctly avoided per 10,000 individuals tested.[3] In a population with T1D, at the same threshold of 10%, the net benefit for MACE in men in SIDIAP was of 2 additional correct interventions while correctly avoiding 5,746 unnecessary interventions for every 10,000 individuals tested (Supplemental Table 4). Especially for T1D, this could become a way forward to aid in decision-making for CVD prevention, especially since no added burden is placed on the healthcare system by utilizing already commonly measured biomarkers, and since the risk profile information seems to be more impactful in T1D than in the general population.

This expansion study leverages robust, large-scale, multi-centre data to validate an established risk profiling framework in a new, clinically significant population, and the inclusion of decision curve analysis further enhances its translational relevance and potential for clinical adoption. Importantly, to define the phenotypic risk profiles, commonly used biomarkers routinely collected during standard medical consultations are used, meaning that the proposed approach would not add any data collection or workload burden for healthcare practitioners. With the exception of waist-to-hip ratio but that remains relatively easy to implement and could be feasibly adopted in future research and/or clinical practice. However, a way of clinically translating the measured biomarkers into the risk profiles is needed, especially by integrating a user-friendly free calculator (https://shiny.gbiomed.kuleuven.be/UMAP_app/) into daily clinical practice, ideally linked to electronic health record systems. Additionally, while the study demonstrated improved CVD and retinopathy prediction in certain populations with T1D, substantial increases in C-statistics are inherently challenging to achieve in already well-calibrated models. Therefore, even small gains in discrimination and decision-curve outcomes can have meaningful implications at the population level, particularly for the early identification and prevention of complications in T1D. On the other hand, the cross-sectional allocation of risk profiles raises uncertainty about their temporal stability, while the stability of the identified phenotypic profiles over time opens the opportunity for future research as longitudinal analyses could provide valuable insights into the temporal consistency of these profiles not only on a population with T1D but also in the general population. Nevertheless, emerging evidence suggests that cross-sectional information can, in some cases, offer predictive performance comparable to—or even exceeding—that of longitudinal data[9,10] while also being more feasible for use in routine clinical practice which is why we believe that this current study still presents benefits to clinical practice. Also, our findings may have limited generalizability given the reliance on predominantly European data with relatively little heterogeneity in ethnicity and health care systems.

References:

1. Harrell F. Statistically Efficient Ways to Quantify Added Predictive Value of New Measurements. Statistical Thinking. https://www.fharrell.com/; 2018.

2. Frank E. Harrell J. Regression Modeling Strategies With Applications to Linear Models, Logistic and Ordinal Regression, and Survival Analysis. 2 ed: Springer Cham; 2015.
3. Coral DE, Smit F, Farzaneh A, et al. Subclassification of obesity for precision prediction of cardiometabolic diseases. *Nature Medicine* 2024.
4. SCORE2 risk prediction algorithms: new models to estimate 10-year risk of cardiovascular disease in Europe. *Eur Heart J* 2021; **42**(25): 2439-54.
5. SCORE2-Diabetes: 10-year cardiovascular risk estimation in type 2 diabetes in Europe. *Eur Heart J* 2023; **44**(28): 2544-56.
6. Zgibor JC, Ruppert K, Orchard TJ, Soedamah-Muthu SS, Fuller J, Chaturvedi N, Roberts MS. Development of a coronary heart disease risk prediction model for type 1 diabetes: the Pittsburgh CHD in Type 1 Diabetes Risk Model. *Diabetes Res Clin Pract* 2010; **88**(3): 314-21.
7. Vistisen D, Andersen GS, Hansen CS, Hulman A, Henriksen JE, Bech-Nielsen H, Jørgensen ME. Prediction of First Cardiovascular Disease Event in Type 1 Diabetes Mellitus: The Steno Type 1 Risk Engine. *Circulation* 2016; **133**(11): 1058-66.
8. Helmink MAG, Hageman SHJ, Eliasson B, et al. Lifetime and 10-year cardiovascular risk prediction in individuals with type 1 diabetes: The LIFE-T1D model. *Diabetes Obes Metab* 2024; **26**(6): 2229-38.
9. Shmatko A, Jung AW, Gaurav K, et al. Learning the natural history of human disease with generative transformers. *Nature* 2025.
10. Rindfleisch A, Malter AJ, Ganesan S, Moorman C. Cross-Sectional versus Longitudinal Survey Research: Concepts, Findings, and Guidelines. *Journal of Marketing Research* 2008; **45**(3): 261-79.

March 2026

Dear Reviewer,

We greatly appreciate your input on our paper. Your valuable suggestions greatly helped fine-tuning the manuscript and we are pleased that the revisions were well received.

We ensure that this article contains only original work. The material in this manuscript has not been published before and is not being considered for publication elsewhere in whole or in part in any language. The conflicts of interest are reported and the copyright assignment form and other required documents will be forthcoming.

On behalf of all authors.
Yours Sincerely,

Bart Van der Schueren, MD, PhD, Prof.
Clinical and Experimental Endocrinology,
Department of Chronic diseases, Metabolism and Ageing
KU Leuven, Belgium

UZ LEUVEN

KU LEUVEN

Reviewers' comments:

Reviewer #4 (Remarks to the Author):

Thank you to the authors for addressing these comments and updating the paper. Your revisions have aligned the findings in the text much more explicitly with the results tables, and the discussion is substantially improved. All my previous concerns have been addressed.

Response:
Thank you for your thoughtful assessment. We are glad to learn that the revisions have resolved the earlier concerns. Your careful constructive feedback throughout this process has been invaluable and greatly improved our work.
